# Sex and out-of-hospital cardiac arrest survival: a systematic review

**DOI:** 10.1186/s13613-022-01091-9

**Published:** 2022-12-19

**Authors:** Ines Lakbar, Mariachiara Ippolito, Aviv Nassiri, Louis Delamarre, Philippe Tadger, Marc Leone, Sharon Einav

**Affiliations:** 1 Aix-Marseille University, Publique Hôpitaux de Marseille, Marseille, France; 2 Department of Anesthesiology and Intensive Care, Hôpital Nord, 13015 Marseille, France; 3 CEReSS, Health Service Research and Quality of Life Centre, School of Medicine – La Timone Medical, Aix-Marseille University, Marseille, France; 4 Department of Surgical, Oncological and Oral Science (Di.Chir.On.S.), University of Palermo, Palermo, Italy; 5 Department of Anaesthesia, Intensive Care and Emergency, Policlinico Paolo Giaccone, Via del Vespro 129, 90127 Palermo, Italy; 6 Department of Military Medicine and Tzameret, Faculty of Medicine, Hebrew University of Jerusalem, Jerusalem, Israel; 7 Medical Corps, Israel Defense Forces, Tel HaShomer, Israel; 8 Real World Evidence, IQVIA, 3600 Genk, Belgium; 9 Intensive Care Unit, Shaare Zedek Medical Center, Jerusalem, Israel

**Keywords:** Sex, Gender disparities, Out-of-hospital cardiac arrest, Outcomes

## Abstract

**Background:**

The literature is unresolved on whether female receive advanced cardiac life support less than do male and on whether female have a survival advantage over male after cardiopulmonary resuscitation.

**Methods:**

We systematically searched PubMed, Embase and Web of Science databases (from inception to 23-April-2022) for papers reporting outcomes in adult male and female after out-of-hospital cardiac arrest. The main study outcome was the rate of adjusted survival to hospital discharge or 30 days. Secondary outcomes included unadjusted survival to hospital discharge and favourable neurological outcome.

**Results:**

A total of 28 studies were included, involving 1,931,123 patients. Female were older than male, their cardiac arrests were less likely to be witnessed and less likely to present with a shockable rhythm. Unadjusted analysis showed that females had a lower likelihood of survival than males (OR 0.68 [0.62–0.74], *I*^*2*^ = 97%). After adjustment, no significant difference was identified between male and female in survival at hospital discharge/30 days (OR 1.01 [0.93–1.11], *I*^*2*^ = 87%). Data showed that male had a significantly higher likelihood of favorable neurological outcome in unadjusted analysis but this trend disappeared after adjustment. Both the primary outcome (adjusted for several variables) and the secondary outcomes were associated with substantial heterogeneity. The variables examined using meta-regression, subgroup and sensitivity analyses (i.e., study type, location, years, population, quality of adjustment, risk of bias) did not reduce heterogeneity.

**Conclusions:**

The adjusted rate of survival to hospital discharge/30 days was similar for male and female despite an initial seeming survival advantage for male. The validity of this finding is limited by substantial heterogeneity despite in-depth investigation of its causes, which raises concerns regarding latent inequalities in some reports nonetheless. Further study on this topic may require inclusion of factors not reported in the Utstein template and in-depth analysis of decision-making processes.

**Supplementary Information:**

The online version contains supplementary material available at 10.1186/s13613-022-01091-9.

## Background

Out-of-hospital cardiac arrest (OHCA), occurs at an incidence of 30.0 to 97.1 individuals per 100,000 people annually [1]. As OHCAs are frequently unexpected yet require immediate treatment, global rates of survival to hospital discharge after OHCA remain dismally low (~ 8.8%) [2]. Patients’ characteristics such as age and co-morbidities affect the outcome [3, 4] but survival is also related to the individual components of the response to OHCA, such as bystander cardiopulmonary resuscitation (CPR) [5–7], early defibrillation [8], targeted temperature management [9, 10] and coronary catheterization [11].

The characteristics of OHCA differ in male and female [12]. Female are usually older [13] and have more co-morbidities (e.g., hypertension, diabetes, obesity) [14–16]. Female arrest in the privacy of their own home and resultantly their arrests tend to be less witnessed [17]. One meta-analysis showed that 77% of female who arrest do so at home vs. 67% of male. Only 40% of OHCAs among female were witnessed vs. 47% among male [18]. In some studies female receive less bystander CPR [17, 19, 20] but this finding is inconsistent [21]. The interval between emergency medical services (EMS) dispatch to EMS–CPR or first rhythm capture may also be longer in female than in male [22]. Possibly as a result of all these, female present less with shockable rhythms than do male [16–18, 23, 24].

It, therefore, seems unsurprising that overall female receive less advanced cardiac life support [25, 26]. Female less frequently undergo defibrillation, receive less epinephrine and are even less likely to undergo endotracheal intubation [18, 22, 27]. There is controversy with regard to whether female are more likely to have return of spontaneous circulation (ROSC) than do male [28] or not [21, 27]. Even when ROSC occurs and the patient is brought to hospital, differences remain in post-resuscitation care as female less frequently undergo coronary angiography [15, 27], percutaneous coronary intervention [16, 26] and other evidence-based interventions [26]. Despite these differences, studies reporting outcomes of male and female after CPR range between better, similar and worse survival outcomes for one sex compared to another.

This systematic review was, therefore, conducted to investigate whether male and female with OHCA have different mortality rates or neurological outcomes despite adjustment for confounding variables.

## Methods

The study was conducted in accordance with the Preferred Reporting Items for Systematic Reviews and Meta-Analyses (PRISMA) recommendations [29, 30] and was registered in the PROSPERO database prior to the study initiation (CRD 42021226050).

### PICO question

Do adult female (P) after out-of-hospital cardiac arrest (I) compared to adult male after out-of-hospital cardiac arrest (C) have different survival rates and neurological outcomes (O)?

### Search strategy

We systematically searched PubMed, Embase and Web of Science databases (inception to 23-April-2022) for papers reporting outcomes at the time of hospital discharge in adult (age 16 years or older) male and female after out-of-hospital cardiac arrest. The search was performed three times to ensure a full and up-to-date review of the literature. No language restriction was applied during the search, then only studies written in English were included. In brief, we used keywords as exact phrases and subject headings according to database syntaxes with the help of an information specialist. The full search strategy is described in Additional file 1.

### Eligibility and inclusion/exclusion criteria

We included papers describing randomised controlled trials, nonrandomised clinical trials, observational cohort studies or case series of adult humans with OHCA. Case reports, animal models and special populations (children, pregnant female) were excluded. Studies were included if sex differences in outcome were their primary study aim and if they evaluated at least one outcome of interest (survival to hospital discharge or 30-day survival). We used author definitions for OHCA. Only studies published after 1995 were included to account for changes after the 1993 declaration of the Council for International Organizations of Medical Sciences regarding inclusion of women in clinical trials.

### Paper selection

The titles and abstracts of all records were screened independently and in duplicate by two of the authors (IL, AN) using the Covidence software tool. The papers selected were downloaded in full and they were reviewed independently and in duplicate by the same two authors to verify fulfilment of inclusion criteria. The reference lists of relevant articles were searched for additional potentially pertinent articles (i.e., snowballing method). All articles rated discrepantly by the two screening authors in the Covidence software were reviewed and discussed one by one by both authors and subsequently included only if both authors agreed on eligibility. In each stage disagreements were resolved by a third author (SE). Among papers reporting completely or partially overlapping data, we selected the paper displaying adjusted data and describing the greatest number of patients. Since many of the included studies presented data from national databases, the risk of overlapping data was high. To avoid such overlap, we excluded any study that presented data from a country whose national database was already being used in another included study on the same period (Additional file 2: Table S1). In this case, the study included was the one with adjusted data, and if more than one study from the same national database had adjusted data, the largest cohort was chosen. Although pre-planned, alternative prioritisation based on clinical sensibility due to poorer adjustment with more patients was never required.

**Fig. 1:**
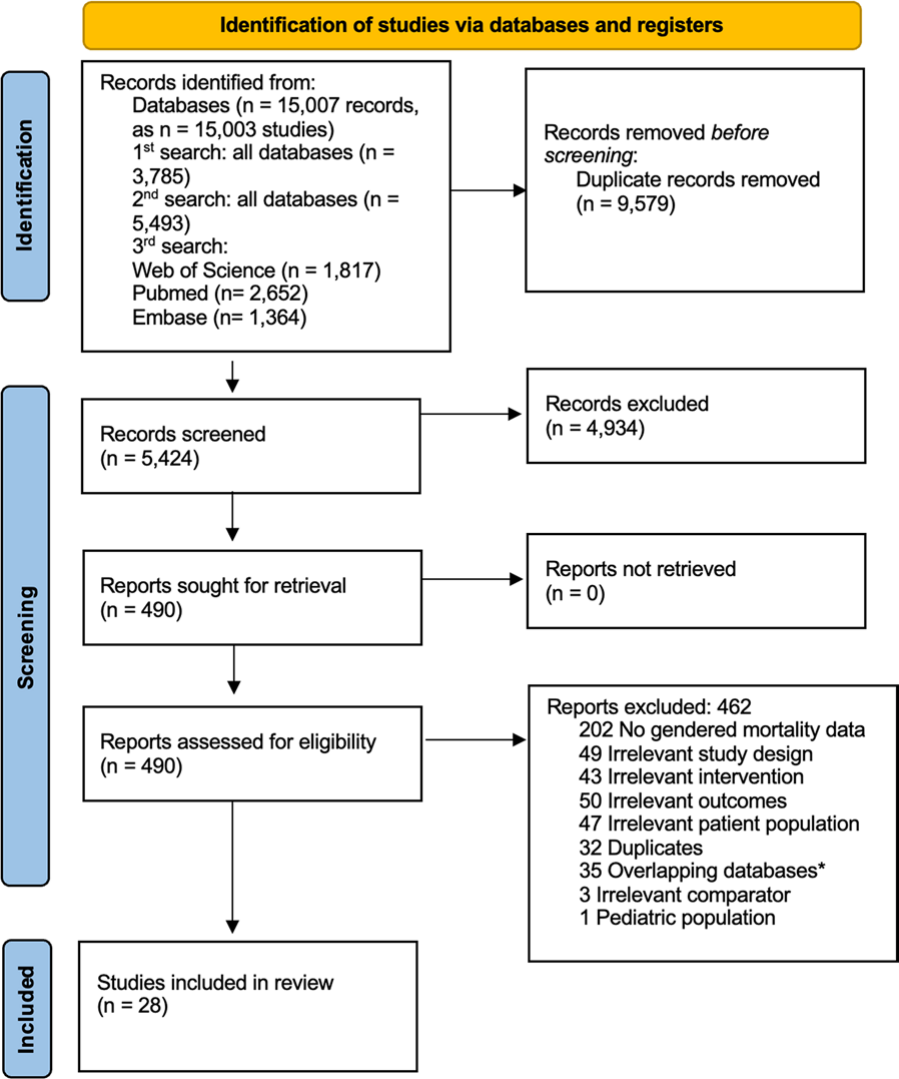
Flow chart of the included studies. *A table summarizing the overlapping databases is provided in the Additional file 2: Table S1)

The list of papers with overlapping populations is presented in Additional file 2. We planned to contact the corresponding authors of the screened studies in case questions arose regarding eligibility or data presentation but no such issues arose. The details of the inclusion/exclusion process are shown in the PRISMA diagram (Fig. 1).

### Data extraction

Two authors (IL, AN) extracted the data in duplicate using a standardized data extraction form. Discrepancies in the extracted data were adjudicated by a third author (SE). The data extracted included study characteristics (e.g., source country, study type, single/multicentre), patient demographics (age, sex), medical background, treatments and outcomes. We also collected data on the type of adjusted analyses performed and the variables adjusted for. The final version of the database was validated by all the investigators involved in data collection (IL, SE, AN) and is available as Additional file 3.

### Assessment of risk of bias

For the primary outcome, two of the authors (IL, MI) assessed the risk of bias (RoB) of the included studies independently and in duplicate using the ROBINS-I tool. [31] Disagreements over RoB were resolved by consensus or, if necessary, adjudicated by a third author (SE).

### Outcomes

The main study outcome was the rate of adjusted survival to hospital discharge or, if not available, at 30 days. Secondary outcomes included the rates of (1) unadjusted survival to hospital discharge (2) favourable neurological outcome at discharge. Favourable neurological outcome at discharge was defined according to Cerebral Performance Category (CPCs) as this is the most commonly used tool for this outcome. CPC 1 and CPC 2 were considered favourable outcomes in our analysis as observed in the studies reporting neurological outcomes.

### Certainty of the evidence

The certainty of the evidence (i.e., the overall effect estimates) was assessed for the primary outcome and the secondary outcomes using Grading of Recommendations Assessment, Development and Evaluation (GRADE) [32].

### Statistical analysis

The population characteristics were described as weighted means and weighted standard deviation for continuous variables; and weighted means of percentages and weighted standard deviation from percentages for categorical variables. For adjusted analyses we used the generic inverse variance method to pool estimates and standard errors (SEs) as per Cochrane guidance [33, 34]. The results were reported as odds ratios (OR) with their 95% confidence intervals (CI) for dichotomous outcomes. A prediction interval (PI) was calculated for the primary outcome and for any outcome with an OR excluding the value of no difference. Meta-analyses were performed using adjusted estimates from multivariate models or propensity-matched cohorts for the mortality outcomes. ORs and CIs were transformed to natural log and SEs using standard formulas. Random effects models were used for all analyses.

We planned sensitivity analyses based on the RoB of the included studies.

Preplanned subgroup analyses were performed to study possible heterogeneity stemming from the number of centres (multi vs. single), study location (Europe, Asia, North America, other), population denominator (i.e., whether non-survivors to hospital admission were accounted for in the cohort or not), OHCA etiology, quality of adjustment variables.

We added an additional subgroup analysis based on the study timeframe after the literature search due to the broad range of years ultimately studied (31 years). The study timeframe was defined as the year of inclusion of the last patient in the cohort rather than the year of publication, since several studies were published many years after completion of patient inclusion. We also intended to perform an analysis separating before/after the 2015 guidelines, allowing for a 1-year implementation period. However, no study included patients strictly from 2016 onward; those most recent pooled data from both periods.

When effect size was attributable to a small number of studies in a subgroup (≤ 5) we calculated a pooled version of τ^2^ to be used across all subgroups, thereby decreasing reliance on an imprecise estimate of between-study heterogeneity in one subgroup [35].

All P values were two-tailed. P values less than 0.05 were considered statistically significant. Statistical heterogeneity (i.e., chance variation between studies) was sought by visual inspection of forest plots and with the nonparametric Cochran’s Q test and the I^2^ statistic [34]. Heterogeneity was considered likely if Q > df (degrees of freedom) and was considered confirmed if the P value was 0.10 or less. The possibility of small-study effects was first explored through inspection of funnel plot. The Harbord’s and Peter’s tests were planned to be performed to investigate small-study effects, except in the case of significant heterogeneity between studies, where an arcsine test using Rücker's random effects was to be preferred, assuming no publication bias [36–38]. These tests were chosen over the Egger’s test, given the dichotomous nature of the outcome of interest [39, 40].

All analyses were performed using R software (R Core Team 2013, R Foundation for Statistical Computing, Vienna, Austria, URL (http://www.R-project.org/) with the package meta [41].

## Results

A total of 5,423 studies were screened, of which 28 were included in the final analysis, corresponding to 1,931,123 patients (1,136,311 male and 794,812 female) (Fig. 1).

### Included studies

All the studies identified were observational and all were retrospective analyses of data from cohorts tracked and documented in real time. Five studies were only published as poster presentations [42–46]. The data covered four continents (Europe, Asia, America and Oceania) over a period of 33 years (1988–2021). There were data from single centre studies [43, 45, 47–49], national registries [20, 21, 25, 44, 46, 50–56] or local emergency medical services registries (e.g., comprised of all OHCAs registered in a city, in several countries or in several countries over a period of time) [17, 42, 57–65].

All studies included only patients with OHCA. Thirteen studies included only OHCA of cardiac etiology [17, 21, 25, 42, 46, 48, 53, 56–59, 63, 65] and fifteen studies included OHCA of both cardiac and non-cardiac etiologies [43–45, 47, 49–52, 54, 55, 60–62, 64]. Among the latter, seven also included patients whose cardiac arrest was due to trauma [20, 49–52, 61, 62] and five did not specify whether traumatic cardiac arrests were excluded or not [43–45, 54, 55]. Twenty-two studies provided survival data from OHCA to discharge (or to day-30), while six studies excluded patients that did not survive to hospital admission, reporting data from hospital admission to discharge (or to day-30) only (Additional file 2: Table S2) [44, 45, 47, 48, 53, 55].

### Baseline data

Female were older than male (weighted means, 71.6 ± 5.1 years vs. 67.4 ± 3.8 years, based on eighteen studies), their arrest was less likely to be witnessed than that of male (weighted means of percentages, 40.5 ± 8.7% vs. 46.0 ± 8.8%, data from 21 studies). Female were more likely to undergo bystander CPR (weighted means of percentages, 46.6 ± 10.7% vs. 40.8 ± 8.9%, data from eighteen studies) but were less likely to present with a shockable rhythm than male (weighted means of percentages, 8.4 ± 4.4% vs. 24.1 ± 12.3%, data from 23 studies).

Less female than male were treated with coronary angiography (weighted means of percentages, 17.8 ± 15.1% vs. 32.3 ± 20.7%, data from four studies), percutaneous coronary intervention (weighted means of percentages, 7.0 ± 7.6% vs. 14.8 ± 7.8%, data from six studies) or targeted temperature management (weighted means of percentages, 12.8 ± 12.2% vs. 17.6 ± 14.6%, data from five studies) (Table 1).

**Table 1:** Description of the included population

Study	Number of patients		% of witnessed OHCA		% of bystander CPR		% of shockable rhythm		% of CAG		% of PCI		% of TTM	
	Male	Female	Male	Female	Male	Female	Male	Female	Male	Female	Male	Female	Male	Female
Ahn [25]	8764	5158	46.94	44.98	–	–	5.36	3.43	–	–	–	–	–	–
Akahane [50]	171,970	104,620	42.13	36.90	32.78	38.31	10.13	4.66	–	–	–	–	–	–
Al-Dury [51]	14,755	7024	–	–	–	–	–	–	–	–	–	–	–	–
Allan [42]	340	110	–	–	–	–	–	–	–	–	–	–	–	–
Arabi [43]	718	269	–	–	–	–	–	–	–	–	–	–	–	–
Arrich [48]	569	205	–	–	–	–	78.21	64.39	–	–	–	–	26.01	23.90
Auricchio [57]	1788	693	70.81	14.57	61.86	57.72	38.26	22.37	–	–	–	–	–	–
Blom [58]	4117	1600	–	–	72.72	67.88	51.66	33.06	25.26	16.44	13.82	8.75	26.18	24.88
Bougouin [47]	1297	520	84.12	80.19	47.11	43.85	60.91	41.92	69.55	49.04	31.61	17.12	–	–
Bray [59]	7345	3108	62.80	57.08	49.26	45.01	45.19	25.03	–	–	–	–	–	–
Castro [44]	533,985	410,175	–	–	–	–	34.30	10.20	–	–	–	–	–	–
Cline [60]	250	138	58.00	43.48	52.00	43.48	53.60	33.33	–	–	–	–	–	–
Dicker [61]	2678	1184	70.09	66.81	–	–	43.05	27.79	–	–	22.03	24.58	–	–
Goto [52]	217,173	169,362	40.27	36.33	49.55	56.45	10.66	4.28	–	–	–	–	–	–
Herlitz [62]	17,149	6648	57.92	55.72	35.37	30.29	32.14	21.13	–	–	–	–	–	–
Hubert [20]	43,655	22,740	66.62	65.67	33.88	33.61	8.73	4.67	–	–	–	–	–	–
Jeong [53]	13,716	6959	64.12	63.03	48.02	48.41	33.53	17.07	–	–	12.06	3.32	11.28	7.46
Johnson [63]	11,745	7653	49.90	44.81	33.72	33.25	28.47	16.50	–	–	–	–	–	–
Kim [64]	7069	3810	54.11	48.32	47.14	44.86	42.79	24.65	–	–	–	–	–	–
Mahapatra [65]	163	37	86.00	82.00	–	–	100	100	–	–	–	–	–	–
Nagraj [45]	80	74	73.8	64.9	33.8	24.3	13.8	10.8	–	–	–	–	36.3	37.8
Ng [21]	24,267	15,892	44.55	37.98	36.61	41.17	17.70	6.70	–	–	–	–	–	–
Pell [54]	15,437	6724	54.95	50.28	–	–	–	–	–	–	–	–	–	–
Perman [55]	3675	2887	–	–	–	–	–	–	18.42	9.32	9.47	3.84	–	–
Rob [49]	693	239	91	84	86	82	65	47	79	63	55	46	86	74
Safdar [17]	7748	3731	48.70	42.80	16.89	12.11	41.60	24.39	–	–	–	–	–	–
Shin [46]	12,111	6934	44.26	41.48	–	–	5.29	2.80	–	–	–	–	–	–
Wissenberg [56]	13,054	6318	50.50	45.08	31.23	23.77	30.49	15.70	–	–	–	–	–	–

*OHCA* out-of-hospital cardiac arrest, *CPR* cardio-pulmonary resuscitation, *CAG* coronary angiography, *PCI* percutaneous coronary intervention, *TTM* Targeted temperature management

### Risk of bias

**Fig. 2:**
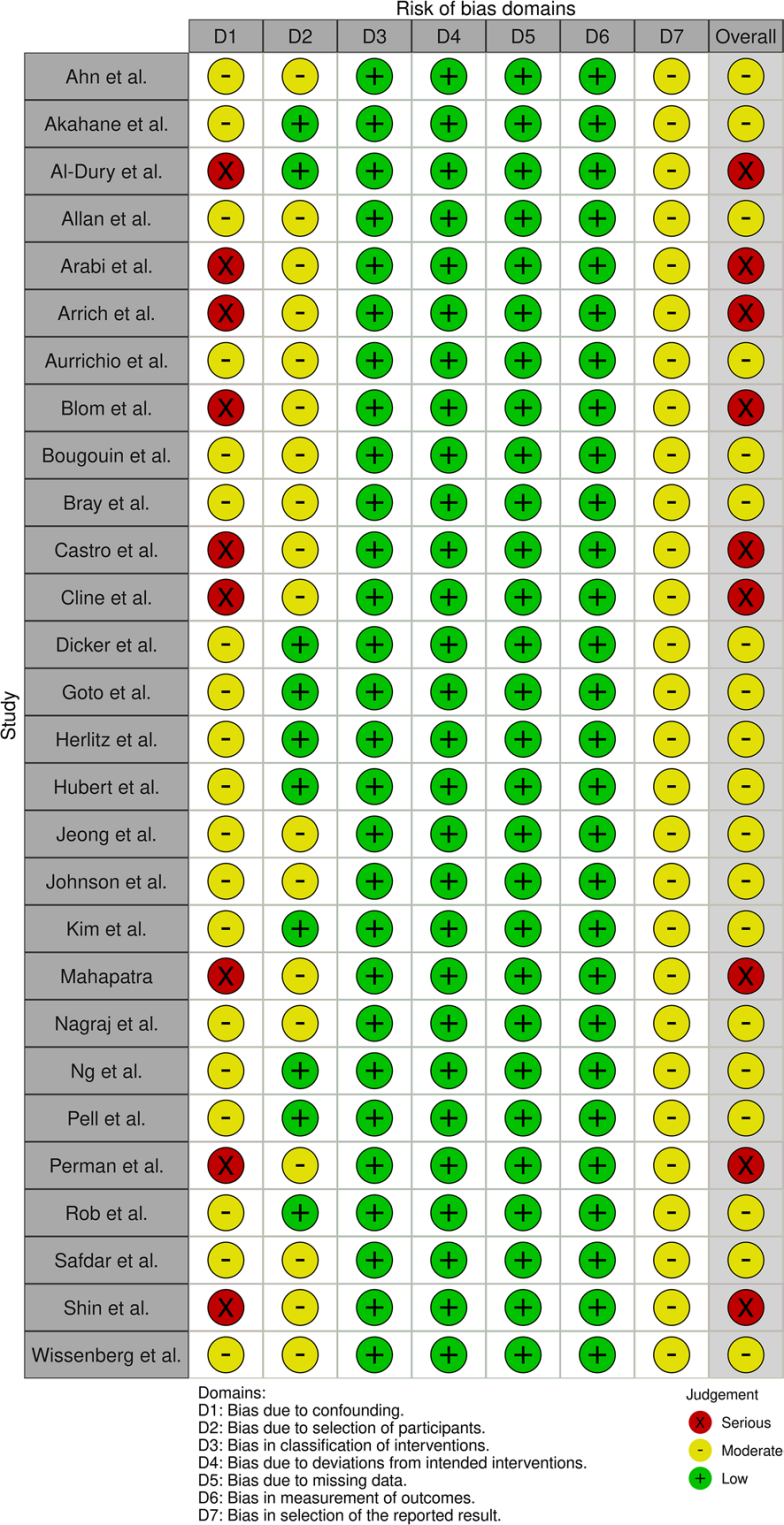
Risk of bias visualisation for the primary outcome: adjusted survival to discharge (or 30-day survival) after OHCA

High RoB was identified predominantly in two domains (Fig. 2); domain 1 which pertains to problems with the adjusted analyses (lack of adjustment and/or lack of important variables in the adjustment) and domain 2 which pertains to selective population inclusion. If only OHCA from cardiac origin or only survivors to hospital admission were included, the domain was rated as moderate at least. The results showed that nine studies were at serious RoB, while the remaining studies were at moderate RoB. The funnel plot suggested publication bias by showing an asymmetry, this was, however, not confirmed by the Rücker’s test (*p* = 0.058).

### Primary outcome

All the studies provided data on survival either to hospital discharge or to day-30 after the occurrence of OHCA. Twenty-one studies provided adjusted data on survival. The variables used for adjustment differed between studies (Table 2).

**Table 2:** Outcomes and variables used for adjustment

Study	Period	Number of patients included in the adjusted analysis		Survival (raw)		Survival timepoint	OR (univariate) as provided by authors		Adjusted survival to hospital discharge as provided by authors		Variables adjusted for
		Male	Female	Male	Female		Reference	OR	Reference	OR	
Ahn [25]	2008	8764	5158	274	94	Hospital discharge	None	None	Female, to discharge	0.82 (0.63–1.05)	Age, location of arrest, witnessed status, presenting rhythm, response time, transport time
Akahane [50]	2005–2007	171,970	104,620	2,587	1,175	30 days	Female, 30 days	1.24 (1.2–1.28)	Female, 30-day survival	1.06 (1.02–1.1)	Age, cause of arrest (cardiac/non cardiac), witnessed status, bystander CPR, defibrillation by EMS, AED by layperson, airway device, epinephrine
Al-Dury [51]	2011–2019	0	0	–	–	30 days	None	None	–	–	–
Allan [42] POSTER	2009–2012	340	110	76	37	Hospital discharge	None	None	Female, to discharge	2.92 (1.44–5.91)	Age, presenting rhythm, witnessed status, bystander CPR, public location, smoking/non-smoking, hypertension, diabetes
Arabi [43] POSTER	1991–2010	0	0	–	–	Hospital discharge	None	None	–	–	–
Arrich [48]	1991–2004	569	205	258	81	Hospital discharge	None	None	Female, to discharge	0.91 (0.60–1.38)	Age, BLS, no-flow time (minutes), low-flow time (minutes), ventricular fibrillation, tachycardia, history of myocardial infarction, history of chronic obstructive pulmonary disease, history of diabetes, history of hypertension, history of cerebral vascular disease, NYHA class (continuous), history of chronic heart failure, smoking, use of therapeutic hypothermia
Auricchio [57]	2002–2018	0	0	–	–	Hospital discharge	None	None	Male, to discharge	1.13 (0.8–1.5)	Age, presenting rhythm, year-groups of OHCA’s occurrence, OHCA location, EMS arrival time, witnessed status and CPR-initiated by laypeople
Blom [58]	2006–2012	0	0	–	–	Hospital discharge	Female, discharge	0.57 (0.48–0.67)	–	–	–
Bougouin [47]	2000–2013	1297	520	442	150	Hospital discharge	None	None	Male, to discharge	0.77 (0.57–1.06)	Age, per year, occurrence at home, shockable rhythm, time from collapse to BLS > 4 min, time from BLS to ROSC > 15 min, epinephrine use, early invasive strategy, post-OHCA shock
Bray [59]	2003–2010	7345	3108	808	218	Hospital discharge	None	None	Female, to discharge	1.11 (0.92–1.33)	Age, witnessed status, bystander CPR, year of arrest, rural location, public location, EMS response time and interaction term if significant, shockable rhythm
Castro [44] POSTER	2012–2016	533,985	410,175	218,934	151,765	Hospital discharge	None	None	Female, to discharge	0.88 (0.86–0.90)	Acute kidney injury, ST–segment elevation myocardial infarction (STEMI), and cardiogenic shock
Cline [60]	1997–1999	250	138	18	3	Hospital discharge	None	None	Female, to discharge	0.29 (0.08–0.99)	Age
Dicker [61]	2013–2015	2,678	1184	432	148	30 days	Male 30-day survival	0.74 (0.61 to 0.91)	Female, 30-day survival	1.22 (0.96–1.55)	Age, location, etiology, initial rhythm, witnessed status
Goto [52]	2013–2016	217,173	169,362	12,373	5,561	30 days	None	None	Male, 30-day survival	1.07 (1.03–1.11)	Age, year, place, witnessed status, presenting rhythm, presumed cause, bystander CPR, airway management, epinephrine, call-to-response time\hospital arrival
Herlitz [62]	1990–2000	17,149	6648	514	199	30 days	None	None	Female, 30-day survival	1.27 (1.03–1.56)	Age, witnessed status, bystander CPR, place of arrest, initial rhythm and etiology
Hubert [20]	2011–2017	43,655	22,740	2,575	978	30 days	Female, 30-day survival	1.29(1.39–1.5)	Female, 30-day survival	0.801(0.697–0.921)	Age, cardiac arrest type, location, bystander presence, bystander type, cardiovascular history, respiratory history, diabetes history, end of life, rhythm
Jeong [53]	2013–2016	13,716	6959	3,795	1,163	Hospital discharge	Female, discharge	0.52 (0.49–0.56)	Female, to discharge	0.87 (0.71–1.08)	Patient-community, EMS factors, comorbidities, metropolitan, place of arrest, witness, bystander CPR, EMS defibrillation, EMS response time, presenting rhythm on scene, time from EMS call to ROSC, hypothermia, level of ED, PCI
Johnson [63]	2005–2009	11,745	7653	1,159	588	Hospital discharge	None	None	Female, to discharge	1.23 (1.09–1.38)	Age, race/ethnicity, public arrest witnessed by bystander vs. by EMS bystander CPR, public AED used shockable rhythm
Kim [64]	1990–1998	7069	3810	1,056	403	Hospital discharge	None	None	Female, to discharge	1.09 (0.93–1.27)	Age, ventricular fibrillation, witnessed status, bystander CPR, categorized location of arrest, response time by first responder, and response time by paramedic
Mahapatra [65]	1990–2000	0	0	–	–	Hospital discharge	None	None	–	–	–
Nagraj [45] POSTER	2019–2021	0	0	–	–	Hospital discharge	None	None	Not provided*	0.84(0.30–2.35)	Unclear
Ng [21]	2009–2012	24,267	15,892	1,936	631	Hospital discharge	Female, to discharge	0.48 (0.43–0.52)	Female, to discharge	0.94 (0.77–1.15)	Age, gender, location type, medical history, witnessed status, bystander CPR, presenting rhythm, prehospital defibrillation, prehospital airway, prehospital drug administration, response time
Pell [54]	1988–1997	15,437	6724	1,113	422	Hospital discharge	None	None	Female, to discharge	0.96 (0.8–1.14)	Age, gender, defibrillation, arrest location, witness, time to CPR
Perman [55]	2010–2011	3675	2887	1,513	1048	Hospital discharge	None	None	Female, to discharge	0.86 (0.77–0.96)	Age, race, and chronic medical conditions
Rob [49]	2012–2020	693	239	369	105	30 days	None	None	Female, 30-day survival	0.98(0.65–1.50)	Age, witnessed status, bystander CPR, presenting rhythm, time of resuscitation, sustained ROSC status on admission, location of arrest, cause of arrest
Safdar [17]	1994–2002	7748	3731	248	63	Hospital discharge	Unclear	Unclear	Female, to discharge	0.88 (0.81–0.96)	Age, witnessed status, presenting rhythm at EMS arrival, bystander CPR, type of ALS (BLS, BLS optimisation, ALS)
Shin 2010 POSTER	2006–2007	0	0	–	–	Hospital discharge	None	None	–	–	–
Wissenberg [56]	2001–2010	0	0	–	–	30 days	Male (above 50), 30 days	0.48 (0.41–0.56)	–	–	–

*OHCA* out-of-hospital cardiac arrest, *CPR* cardio-pulmonary resuscitation, *EMS* emergency medical services, *ALS* advanced life support, *BLS* basic life support, *ROSC* return of spontaneous circulation, *AED* automated external defibrillator

^*^Adjusted results provided but no information on whether male or female are comparator

**Fig. 3:**
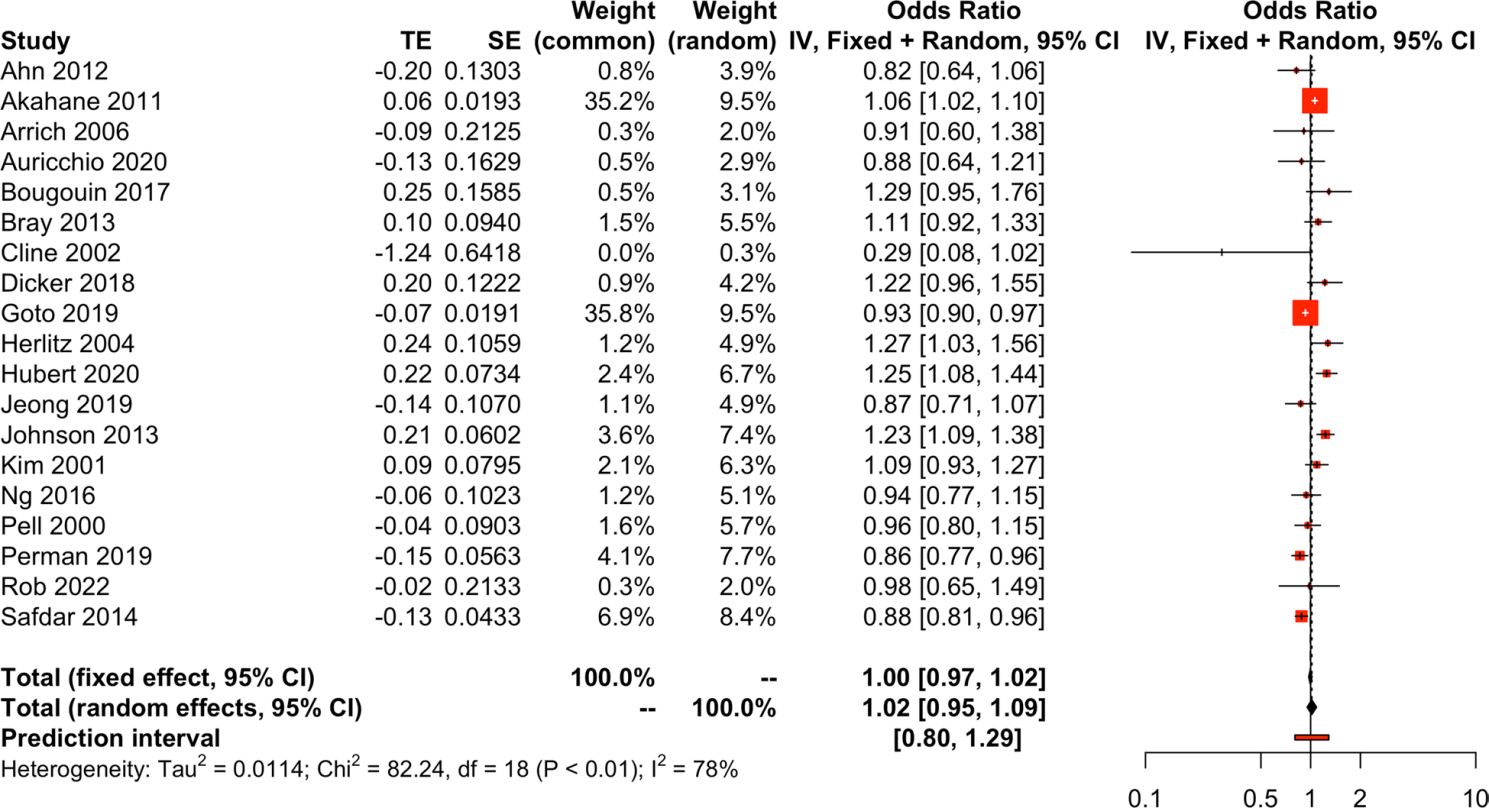
Forest plot of adjusted data on male and female survival to discharge or to 30 days after OHCA

While unadjusted data showed a lower likelihood of survival in females than in males, with an OR 0.68 [0.62–0.74], PI [0.43; 1.05] (*I*^*2*^ = 97%), adjusted aggregated data showed no difference in survival between male and female OR 0.98 [0.92–1.05], PI [0.76–1.35] (Fig. 3). As heterogeneity was high for our primary outcome (*I*^*2*^ = 86%, Fig. 3), sub-group and meta-regression analyses were performed as preplanned. Subgroup meta-analyses based on the quality of the variables adjusted for, geographical location, etiology of OHCA, type of cohort, number of centres and population denominator (Additional file 4) and meta-regression analyses for the same variables (Additional file 4) did not reduce heterogeneity. The quality of the variables adjusted for was added as a post-hoc analysis, as the latter was very uneven. Sensitivity analysis omitting studies identified as outliers most affecting heterogeneity, studies that had included traumatic arrest as the cause of OHCA or omitting studies assessed as having high RoB (Additional file 4) also did not reduce heterogeneity. We also tried to omit studies displaying data in a non-Utstein style, with no satisfactory results. Publication bias was also not found (Additional file 4). In other words, survival to hospital discharge or 30 days was not significantly associated with any of the variables that could be adjusted for when using published data (Table 3).

**Table 3:** Results of meta-analyses

Type of analysis	Subgroup	Random effect model (odds ratio and 95% confidence interval)	Heterogeneity	
			*I*^*2*^	*p value*
Primary outcome meta-analysis: adjusted survival to discharge or to 30 days after OHCA	None	1.01 [0.93; 1.11]–PI [0.76; 1.35]	87%	< 0.01
Secondary outcome meta-analysis: unadjusted survival to discharge or to 30 days after OHCA	None	0.68 [0.62; 0.74]–PI [0.43–1.05]	97%	< 0.01
Secondary outcome meta-analysis: unadjusted neurological intact survival	None	0.56 [0.49; 0.66]–PI [0.32; 0.98]	95%	< 0.01
Secondary outcome meta-analysis: adjusted neurological intact survival	None	0.96 [0.83; 1.10]–PI [0.63; 1.46]	84%	< 0.001
Subgroup analysis by quality of variables of adjustment (as defined in the manuscript)	Strong variables (17 studies)	1.05 [0.98; 1.12]–PI [0.82; 1.34]	80%	< 0.001
	Medium variables (3 studies)	0.84 [0.68; 1.04]–PI [0.12; 6.07]	32%	0.23
	Weak variables (1 study)	0.88 [0.71; 1.09]	Not applicable	
Subgroup analysis by geographical location	Asia (5 studies)	0.94 [0.84; 1.06]–PI [0.63; 1.41]	85%	< 0.01
	North America (7 studies)	0.98 [0.88; 1.09]–PI [0.71; 1.34]	88%	< 0.01
	Europe (7 studies)	1.10 [0.97; 1.25]–PI [0.79; 1.53]	45%	0.09
	Oceania (2 studies)	1.16 [0.93; 1.43]	0%	0.54
Subgroup analysis by OHCA etiology	Cardiac etiology (9 studies)	0.99 [0.86; 1.14]–PI [0.64; 1.55]	77%	< 0.001
	All etiologies (12 studies)	1.02 [0.95; 1.11]–PI [0.79; 1.32]	90%	< 0.001
Subgroup analysis by type of cohort and number of centres	National databases (8 studies)	0.95 [0.87; 1.04]–PI [0.71; 1.28]	92%	< 0.001
	Local cohort from EMS (9 studies)	1.10 [0.99; 1.22]–PI [0.82; 1.47]	81%	< 0.001
	Monocentric (3 studies)	1.08 [0.84; 1.39]–PI [0.13; 9.19]	5%	0.35
	International cohort from EMS (1 study)	0.94 [0.70; 1.26]	Not applicable	
Subgroup analysis by population denominator	All OHCA (15 studies)	1.05 [0.97; 1.13]–PI [0.81; 1.35]	81%	< 0.01
	Only survivors to admission (5 studies)	0.89 [0.83; 0.96]–PI [0.74; 1.08]	34%	0.20
Sensitivity analysis omitting papers rated as outliers (in the Baujat plot)	None	0.97 [0.91; 1.04]–PI [0.80; 1.17]	55%	< 0.01
Sensitivity analysis omitting papers including data of traumatic etiology for OHCA	None	1.01 [0.90; 1.15]–PI [0.68; 1.51]	75%	< 0.01
Sensitivity analysis of papers assessed with high risk of bias	None	1.05 [0.98; 1.12]–PI [0.82; 1.34]	80%	< 0.01

*OHCA* out-of-hospital cardiac arrest, *EMS* emergency medical services

For subgroups analyses, only pooled *τ*^*2*^ results are presented

The pre-planned strategy was to refrain from aggregating data if heterogeneity was high. After seeking clinical heterogeneity that might have explained the levels of statistical heterogeneity, we concluded that the latter was stemming from latent variables and we decided to share the meta-analyses as supplemental data to show our honest reasoning and approach to the reader.

## Secondary outcomes

The meta-analyses performed for the secondary outcomes are reported in Additional file 4. Females had a lower likelihood of unadjusted survival than males with an OR 0.68 [0.62–0.74], PI [0.43; 1.05] (*I*^*2*^ = 97%), and females also had a significantly lower likelihood of favourable neurological outcome than males with an OR 0.56 [0.49–0.66], PI [0.32; 0.98], (*I*^*2*^ = 95%). This trend disappeared when the data were adjusted, without difference between male in female in adjusted neurologically intact survival with an OR 0.96 [0.83–1.10], PI [0.63; 1.46] (*I*^*2*^ = 84%).

We found high heterogeneity (ranging from *I*^*2*^ = 98% to *I*^*2*^ = 83%) for unadjusted survival to discharge/30 days, and both adjusted and unadjusted favourable neurological outcome, precluding any interpretation.

## Certainty of the evidence

The results of the GRADE assessment with regard to primary and secondary outcomes are reported in e-Table 3. Certainty was rated as low for the estimated rates of adjusted and unadjusted survival to hospital discharge (or 30-day survival), and low for the adjusted rates of neurological outcomes.

## Discussion

This analysis identified no difference between male and female in the adjusted rate of survival to hospital discharge or 30 days after OHCA. This finding is striking when compared to the major survival advantage for male in our unadjusted analysis. Some of the unadjusted difference in the outcomes of male and female may be explained by baseline factors less conducive to a successful outcome (i.e., older age, greater comorbidity, less witnessed arrests and less shockable rhythms). This finding is in line with the "gender paradox" described by Bougouin et al., wherein females have similar survival outcomes despite worse prognostic factors than males [18]. Adjustment for many of these factors seems to have corrected the initial imbalance. However, the ongoing heterogeneity in our meta-analyses for both the primary and secondary outcomes suggests prudence is still required before equal outcomes are assumed. Had some of our adjusted analyses shown less heterogeneity, this would have served as proof that ultimate survival is related to the factors studied. Our failure to eliminate or even reduce heterogeneity suggests the presence of latent factors that could still tilt the final balance in favor of better survival for either male or female. This latent factors could include disparities in post resuscitation care, such as access to CAG, PCI and TTM as suggested in our data. These elements were not included as variables for adjustment in many studies and may relate to upstream factors or inequities in the system of care. Post-resuscitation management has been reported to be more conservative in women than in men (less referral for cardiac interventions in particular) [12]. We found no sex differences in survival despite the fact that females have worse prognostic factors than males. Therefore, the sex-related risk–benefit ratios of specific treatments should undergo thoughtful reconsideration as there may be unwarranted inequalities in the care we provide to our patients.

Three metanalyses have been published on the topic of sex differences in outcomes following OHCA. One was published in 2015 and, therefore, required an update [18]. We identified and included fifteen papers published after the last search date of this study. The second was published more recently but suffers from several major flaws; its protocol was not registered, sex differences as primary and secondary outcomes were pooled, there was no division of single centre, national registry and emergency medical services data, changes over time were not studied and, most importantly, adjusted and unadjusted outcomes were not separated [66]. The third meta-analysis was performed on adjusted data, but it too was not registered and its main focus was on age as a confounder [13]. All of these meta-analyses reported a high degree of heterogeneity as do we. The authors of one of these meta-analyses have put forward that differences in survival of male and female may be “a matter of education" (i.e., poorer outcomes in locations with greater inequality for female) [66]. We sought such effects in the subgroup analysis by geographical location and found none. Put together, all of these findings suggest the need to study variables outside those reported in the Utstein template. Our failure to elucidate the causes of heterogeneity suggests the presence of unstudied factors (e.g., in-hospital treatment and/or decision making). Ignoring this finding may perpetuate latent inequalities, perhaps even unrelated to sex.

We did find important selection bias introduced by use of a population denominator of survivors to hospital admission. While this bias seems to contribute little to the heterogeneity observed in relation to our PICO question, it may affect other studies related to survival outcomes. The omission of some cases is often understandable in emergency circumstances. However, our finding suggests there is a need to establish a reporting template that highlights inclusion bias when reporting outcomes after cardiopulmonary resuscitation.

The differences observed between males and females in this analysis may be explained by multiple factors. Females clearly have worse predisposing factors than males at the time of cardiac arrest. In some places poor female education may lead to neglect of risk factors and even late referral if symptoms occur pre-arrest. There may be intrinsic differences in physiological responses to ischemia–reperfusion mechanisms which require elucidation. Finally post-resuscitation care may differ based on family request, professional concerns regarding potential risk/benefit ratio and more.

Our study has several strengths. We prospectively registered the study protocol, performed analysis of adjusted data, assessed the RoB of the included studies and applied GRADE to assess the certainty of the evidence. However, any meta-analysis is only as good as the studies it includes. Most of the studies included in this review were judged at high RoB and neither randomization nor blinding are an option with regard to the study question at hand. Analysis of retrospective studies (regardless of real time data collection) carries an inherent risk of confounding due to study design. Consequently, GRADE, which depends on the lowest quality of evidence for the clinically meaningful outcomes being studied, yielded only low certainty for our conclusion. This is further compounded by the fact that our strategy to handle statistical heterogeneity was unsuccessful. The internal and external validity of the studies included are low and the study populations are also very heterogeneous. Finally, although we studied adjusted data, the variables adjusted for differed between studies. Some studies did adjust their data on care management variables (speed of intervention, PCI, TTM), and there was a possibility of collinearity between these variables and sex, as differences in care themselves can be related to sex leading to a risk of overfitting in our analysis. Sensitivity analyses excluding those studies did not lead to a significant change in results.

## Conclusions

No difference was found between male and female in the adjusted rate of survival to hospital discharge or at 30 days after OHCA. Substantial heterogeneity and failure to elucidate its causes suggest the existence of undetermined factors that were not reported in the Utstein template or the underestimation of factors, such as post-resuscitation care. This systematic review calls for additional further investigation for possible latent inequalities in some reports. Future studies should include data on post-resuscitation care (such as provision of CAG, PCI and TTM) as well as information that is currently no included in the Utstein template such as end of life decisions and in-depth analysis of decision-making processes with regard to provision of organ and life support.

## Supplementary Information

**Additional file 1: **Full search strategy.

**Additional file 2: Table S1. Overlapping studies: **To avoid overlap, we excluded any study that presented data from a country whose national database was already being used in another included study on the same period. In this case, the study included was the one with adjusted data, and if more than one study from the same national database had adjusted data, the largest cohort was chosen. **Table S2.** Description of the population according to the inclusion and exclusion criteria of each study. **Table S3. **Certainty estimates of different outcomes using Grading of Recommendations Assessment, Development and Evaluation (GRADE) methods. **Table S4 **Meta-regression results.

**Additional file 3: **Database.

**Additional file 4: Figure S3. **Quality of the variables used for adjustment: subgroup analysis of adjusted data on men and women survival to discharge or to 30 days after OHCA. **Figure S3. BIS. **Quality of the variables used for adjustment: subgroup analysis of adjusted data on men and women survival to discharge or to 30 days after OHCA (*with the pooled τ*^*2*^). **Figure S4. **Geographical location: subgroup analysis of adjusted data on men and women survival to discharge or to 30 days after OHCA. **Figure S4 BIS. **Geographical location: subgroup analysis of adjusted data on men and women survival to discharge or to 30 days after OHCA (*with the pooled τ*^*2*^).** Figure S5. **OHCA etiology: subgroup analysis of adjusted data on men and women survival to discharge or to 30 days after OHCA. **Figure S6. **Type of cohort and number of centres: subgroup analysis of adjusted data on men and women survival to discharge or to 30 days after OHCA. **Figure S6. BIS **Type of cohort and number of centres: subgroup analysis of adjusted data on men and women survival to discharge or to 30 days after OHCA (*with the pooled τ*^*2*^). **Figure S7. **Population denominator (whether non-survivors to hospital admission were included or not): subgroup analysis of adjusted data on men and women survival to discharge or to 30 days after OHCA.** Figure S8. **Scatter plot showing the results of the regression of time vs. the log(OR) for adjusted survival to discharge or to 30 days. A log(OR) below 0 favors men and a log(OR) above 0 favors women. **Figure S9. A **Baujat plot showing the contribution of each study to the overall heterogeneity. **B **Same plot zoomed in Studies are numbered in alphabetical order. **Figure S10**. Sensitivity analysis omitting studies rated identified as outliers in the Baujat plot (see Fig. 9)—adjusted survival data. **Figure S11**. Sensitivity analysis omitting studies including traumatic etiology for OHCA—adjusted survival data. **Figure S12**. Sensitivity analysis omitting studies assessed as having a high risk of bias. **Figure S13. **Forest plot of unadjusted data on men and women survival to discharge or to 30 days after OHCA. **Figure S14.** Funnel plot of the included studies. **Figure S15. **Forest plot of unadjusted data on men and women neurologically intact survival. **Figure S16.** Forest plot of adjusted data on men and women favourable neurological outcome in survival

## Data Availability

Data are available in the literature corpus and can be find using the search strategy defined in the manuscript.
