# Supplementary material for: Sex and out-of-hospital cardiac arrest survival: a systematic review
Source: Ann Intensive Care. 2022 Dec 19;12:114. doi: 10.1186/s13613-022-01091-9 (PMC9763524; doi:10.1186/s13613-022-01091-9)
Supplement: Supplementary file 1 — Additional file 1: Full search strategy. [file 13613_2022_1091_MOESM1_ESM.docx]

**Additional file 1 – Full search strategy**

**Web of science:** TS=((('cardiac arrest'/exp OR 'cardiac arrest' OR 'myocardial infraction') AND ('gender'/exp OR 'gender' OR 'gender'/exp OR gender OR 'sex'/exp OR 'sex' OR 'sex'/exp OR sex) AND ('outcome'/exp OR 'outcome' OR 'outcome'/exp OR outcome OR 'survival'/exp OR 'survival' OR 'survival'/exp OR survival OR 'mortality'/exp OR 'mortality' OR 'mortality'/exp OR mortality)))

**Embase:** (**'out-of-hospital cardiac arrest'**/exp OR **'out-of-hospital cardiac arrest'**) AND (**'gender'** OR **'gender'**/exp OR **gender** OR **'sex'** OR **'sex'**/exp OR **sex**) AND (**'outcome'** OR **'outcome'**/exp OR **outcome** OR **'survival'** OR **'survival'**/exp OR **survival** OR **'mortality'** OR **'mortality'**/exp OR **mortality**)

**Pubmed: ('cardiac arrest') and (gender OR sex) and (outcome OR survival OR mortality)**

("heart arrest"[MeSH Terms] OR ("heart"[All Fields] AND "arrest"[All Fields]) OR "heart arrest"[All Fields] OR ("cardiac"[All Fields] AND "arrest"[All Fields]) OR "cardiac arrest"[All Fields]) AND ("gender identity"[MeSH Terms] OR ("gender"[All Fields] AND "identity"[All Fields]) OR "gender identity"[All Fields] OR "gendered"[All Fields] OR "gender s"[All Fields] OR "gendering"[All Fields] OR "genderized"[All Fields] OR "genders"[All Fields] OR "sex"[MeSH Terms] OR "sex"[All Fields] OR "gender"[All Fields] OR ("sex"[MeSH Terms] OR "sex"[All Fields])) AND ("outcome"[All Fields] OR "outcomes"[All Fields] OR ("mortality"[MeSH Subheading] OR "mortality"[All Fields] OR "survival"[All Fields] OR "survival"[MeSH Terms] OR "survivability"[All Fields] OR "survivable"[All Fields] OR "survivals"[All Fields] OR "survive"[All Fields] OR "survived"[All Fields] OR "survives"[All Fields] OR "surviving"[All Fields]) OR ("mortality"[MeSH Terms] OR "mortality"[All Fields] OR "mortalities"[All Fields] OR "mortality"[MeSH Subheading]))
