# Supplementary material for: Sex and out-of-hospital cardiac arrest survival: a systematic review
Source: Ann Intensive Care. 2022 Dec 19;12:114. doi: 10.1186/s13613-022-01091-9 (PMC9763524; doi:10.1186/s13613-022-01091-9)
Supplement: Supplementary file 2 — Additional file 2: Table S1. Overlapping studies: To avoid overlap, we excluded any study that presented data from a country whose national database was already being used in another included study on the same period. In this case, the study included was the one with adjusted data, and if more than one study from the same national database had adjusted data, the largest cohort was chosen. Table S2. Description of the population according to the inclusion and exclusion criteria of each study. Table S3. Certainty estimates of different outcomes using Grading of Recommendations Assessment, Development and Evaluation (GRADE) methods. Table S4 Meta-regression results. [file 13613_2022_1091_MOESM2_ESM.docx]

**Additional file 2: Table S1. Overlapping studies:** To avoid overlap, we excluded any study that presented data from a country whose national database was already being used in another included study on the same period. In this case, the study included was the one with adjusted data, and if more than one study from the same national database had adjusted data, the largest cohort was chosen.

| **Location** | **Authors -overlapping** | **Timeline** | **Population** | **Adjusted data** | **Inclusion in the analysis** |
| --- | --- | --- | --- | --- | --- |
| USA national registry | Castro 2019 | 2012-2016 | 944160 | Provided | Yes |
| USA - Specific counties | Cline 2002 | 1997-1999 | 388 | Provided | Yes |
| USA - Several cities | Johnson 2013 | 2005-2009 | 19398 | Provided | Yes |
| USA - Several counties | Kim 2001 | 1990-1998 | 10879 | Provided | Yes |
| USA- Monocentric | Nagraj 2022 | 2019-2021 | 154 | Performed but not provided | Yes |
| USA - State’s registry | Mahapatra 2003 | 1990-2000 | 200 | Not provided | Yes |
| USA - State’s registry | Perman 2019 | 2010-2011 | 6562 | Provided | Yes |
| USA national registry | Akinyele 2016 – Overlaps Castro 2019 | 2002-2013 | 154177 | Provided | No |
| USA national registry | Arunachalam 2020 – Overlaps Castro 2019 | 2012-2013 | 85988 | Provided | No |
| USA national registry | Awad 2021- Overlaps Castro 2019 | 2011-2015 | 23725 | Provided | No |
| USA national registry | Awad 2021 – Overlaps Castro 2019 | 2011-2016 | 8115 | Not provided | No |
| USA national registry | Awad 2021 – Overlaps Castro 2019 | 2011-2016 | 23725 | Provided | No |
| USA national registry | Blewer 2018 – Overlaps Castro 2019 | 2011-2015 | 19331 | Provided | No |
| USA - City’s registry | Bosson 2016 – Overlaps Castro 2019 | 2011-2014 | 5174 | Not provided | No |
| USA national registry | Grunau 2020 – Overlaps Castro 2019 | 2011-2015 | 61473 | Not provided | No |
| USA national registry | Karlsson 2015 – Overlaps Castro 2019 | 2006-2012 | 1667 | Provided | No |
| USA national registry | Kotini-Shah 2021 – Overlaps Castro 2019 | 2013-2019 | 326138 | Provided | No |
| USA national registry | Kotini-Shah 2018 – Overlaps Castro 2019 | 2013-2016 | 7765 | Provided | No (abstract of the article above Kotini-Shah 2021) |
| USA national registry | MaltaHansen 2018 – Overlaps Castro 2019 | 2010-2014 | 8090 | Not provided | No |
| USA national registry | Mody 2021 – Overlaps Castro 2019 | 2011-2015 | 4875 | Provided | No |
| USA national registry | Morris 2021 – Overlaps Castro 2019 | 2013-2019 | 123419 | Not provided | No |
| USA national registry | Morrison 2016 – Overlaps Johnson 2013 | 2005-2007 | 14690 | Provided | No |
| USA national registry | Patel 2016 – Overlaps Castro 2019 | 2000-2013 | 444342 | Provided | No |
| USA national registry | Rosenblatt 2022 – Overlaps Castro 2019 | 2011-2015 | 50644 | Provided | No |
| USA - State’s registry | Teodorescu 2012– Overlaps Johnson 2013 | 2002-2007 | 1296 | Provided | No |
| USA national registry | Vogelsong 2021 – Overlaps Castro 2019 | 2012-2017 | 2407 | Provided | No |
| Sweden’s national registry | Al-Dury 2020 | 2011-2019 | 21779 | Not provided | Yes |
| Sweden’s national registry | Herlitz 2004 | 1990-2000 | 23797 | Provided | Yes |
| Sweden’s national registry | Adielsson 2011 - overlaps with Herlitz | 1990-2009 | 7187 | Provided | No |
| Sweden’s national registry | Perers 1999 – overlaps with Herlitz | 1980-1996 | 3401 | Provided | No |
| Sweden’s national registry | Banck 2013 – overlaps with Al-Dury | 2008-2012 | 2350 | Provided | No |
| Sweden’s national registry | Lindgren 2015 -overlaps with Al-Dury | 2008-2013 | 2589 | Not provided | No |
| Sweden’s national registry | Lindgren 2019 - overlaps with Al-Dury | 2008-2013 | 1498 | Not provided | No |
| Japan’s national registry | Akahane 2011 | 2005-2007 | 276590 | Provided | Yes |
| Japan’s national registry | Goto 2019 | 2013-2016 | 386535 | Provided | Yes |
| Several South-east Asia countries – including Japan and south-korea | Ng 2016 | 2009-2012 | 40159 | Provided | Yes |
| Japan’s national registry | Hagihara 2017 – overlaps with Akahane and Ng | 2005-2012 | 25431 | Not provided | No |
| Japan’s national registry | Kamikura 2017 - overlaps with Akahane and Ng | 2009-2014 | 82197 | Provided | No |
| Japan’s national registry | Kitamura 2010 - overlaps with Akahane | 1998-2007 | 26940 | Provided | No |
| South Korea’s national registry | Ahn 2012 | 2008 | 13922 | Provided | Yes |
| South Korea’s national registry | Jeong 2019 | 2013-2016 | 20675 | Provided | Yes |
| South Korea’s national registry | Shin 2010 | 2006-2007 | 19045 | Not provided for outcomes of interest | Yes |
| South Korea’s national registry | Ahn 2019 – overlaps with Ahn and Ng | 2008-2015 | 121900 | Provided | No (abstract that overlaps with the main paper Ahn 2012, no details in the data) |
| South Korea’s national registry | Hwang 2021 - overlaps with Ahn, Ng and Jeong | 2008-2015 | 121900 | Not provided | No |
| South Korea’s national registry | Song 2022- overlaps with Jeong | 2015-2020 | 6424 | Provided | No |
| South Korea’s national registry | Oh 2017 - overlaps with Ahn and Ng | 2007-2012 | 930 | Provided | No |
| France - Single center | Bougouin 2017 | 2000-2013 | 1817 | Provided | Yes |
| France’s national registry | Hubert 2020 | 2011-2015 | 66395 | Provided | Yes |
| France’s national registry | Karam 2016 – overlaps with Hubert | 2011-2017 | 12426 | Not provided | No |
| Single center in Doha | Arabi 2013 | 1991-2010 | 987 | Not provided | Yes |
| Doha, Qatar registry | Omosola 2018 | 1991-2012 | 496 | Not provided | No |
| Denmark’s national registry | Wissenberg 2014 | 2001-2010 | 19372 | Provided | Yes |
| Copenhagen’s registry | Winter-Jensen 2018 | 2007-2011 | 704 | Provided | No |
| Switzerland – regional registry | Auricchio 2020 | 2002-2018 | 2481 | Provided | Yes |
| Switzerland – regional registry | Baldi 2019 | 2015-2017 | 2076 | Not provided | No |
| Czech Republic –  Regional registry | Rob 2022 | 2012-2020 | 932 | Provided | Yes |

**Additional file 2: Table S2.** Description of the population according to the inclusion and exclusion criteria of each study

| **Study** | **Dead before admission included** | **Cardiac etiology** | **Traumatic etiology** |
| --- | --- | --- | --- |
| **Ahn 2012** | Patients dead in the pre-hospital setting were included | Cardiac etiology only | No trauma patients included |
| **Akahane 2011** | Patients dead in the pre-hospital setting were included | Cardiac and non-cardiac etiologies | Trauma patients included |
| **Al-Dury 2020** | Patients dead in the pre-hospital setting were included | Cardiac and non-cardiac etiologies | Trauma patients included |
| **Allan 2016** | Patients dead in the pre-hospital setting were included | Cardiac etiology only | No trauma patients included |
| **Arabi 2013** | Unknown | Cardiac and non-cardiac etiologies | Unknown |
| **Arrich 2006** | Patients dead in the pre-hospital setting were not included | Cardiac etiology only | No trauma patients included |
| **Auricchio 2020** | Patients dead in the pre-hospital setting were included | Cardiac etiology only | No trauma patients included |
| **Blom 2019** | Patients dead in the pre-hospital setting were included | Cardiac etiology only | No trauma patients included |
| **Bougouin 2017** | Patients dead in the pre-hospital setting were not included | Cardiac and non-cardiac etiologies | No trauma patients included |
| **Bray 2013** | Patients dead in the pre-hospital setting were included (two models provided, we selected the one including the patients who died in the pre-hospital setting) | Cardiac etiology only | No trauma patients included |
| **Castro 2019** | Patients dead in the pre-hospital setting were not included | Cardiac and non-cardiac etiologies | Unknown |
| **Cline 2002** | Patients dead in the pre-hospital setting were included | Cardiac and non-cardiac etiologies | No trauma patients included |
| **Dicker 2018** | Patients dead in the pre-hospital setting were included | Cardiac and non-cardiac etiologies | Trauma patients included |
| **Goto 2019** | Patients dead in the pre-hospital setting were included | Cardiac and non-cardiac etiologies | Trauma patients included |
| **Herlitz 2004** | Patients dead in the pre-hospital setting were included | Cardiac and non-cardiac etiologies | Trauma patients included |
| **Hubert 2020** | Patients dead in the pre-hospital setting were included | Cardiac and non-cardiac etiologies | Trauma patients included |
| **Jeong 2019** | Patients dead in the pre-hospital setting were not included | Cardiac etiology only | No trauma patients included |
| **Johnson 2013** | Patients dead in the pre-hospital setting were included | Cardiac etiology only | No trauma patients included |
| **Kim 2001** | Patients dead in the pre-hospital setting were included | Cardiac and non-cardiac etiologies | No trauma patients included |
| **Mahapatra 2003** | Patients dead in the pre-hospital setting were included | Cardiac etiology only | No trauma patients included |
| **Nagraj 2022** | Patients dead in the pre-hospital setting were not included | Cardiac and non-cardiac etiologies | Unknown |
| **Ng 2016** | Patients dead in the pre-hospital setting were included | Cardiac etiology only | No trauma patients included |
| **Pell 2000** | Patients dead in the pre-hospital setting were included | Cardiac and non-cardiac etiologies | Unknown |
| **Perman 2019** | Patients dead in the pre-hospital setting were not included | Cardiac and non-cardiac etiologies | Unknown |
| **Rob 2022** | Patients dead in the pre-hospital setting were included | Cardiac and non-cardiac etiologies | Trauma patients included |
| **Safdar 2014** | Patients dead in the pre-hospital setting were included | Cardiac etiology only | No trauma patients included |
| **Shin 2010** | Patients dead in the pre-hospital setting were included (based on the description of the CAVAS project in the literature) | Cardiac etiology only | No trauma patients included |
| **Wissenberg 2014** | Patients dead in the pre-hospital setting were included | Cardiac etiology only | No trauma patients included |

**Additional file 2: Table S3.** Certainty estimates of different outcomes using Grading of Recommendations Assessment, Development and Evaluation (GRADE) methods

| **Certainty assessment** | | | | | | | **Effect**  **(Odds ratio 95% CI)** | **Certainty** | **Importance** |
| --- | --- | --- | --- | --- | --- | --- | --- | --- | --- |
| **Number of studies** | **Study design** | **Risk of bias** | **Inconsistency** | **Indirectness** | **Imprecision** | **Other considerations** |  |  |  |
| *Unadjusted survival to discharge* | | | | | | | | | |
| 28 | Observational studies | Not serious | Serious ^a^ | Not serious | Not serious | None | OR= 0.68 (0.62-0.74) | ⨁⨁◯◯  LOW | Critical |
| *Adjusted survival to discharge* | | | | | | | | |  |
| 21 | Observational studies | Not serious | Serious ^a^ | Not serious | Not serious | None | OR= 0.98 (0.92-1.05) | ⨁⨁◯◯  LOW | Critical |
| *ROSC* | | | | | | | | | |
| 9 | Observational studies | Serious | Serious ^a^ | Not serious | Not serious | None | OR= 0.91 (0.77-1.08) | ⨁◯◯◯  VERY LOW | Critical |
| *Adjusted neurological intact survival* | | | | | | | | | |
| 7 | Observational studies | Not Serious | Serious ^a^ | Not serious | Not serious | None | OR= 0.93 (0.81-1.07) | ⨁⨁◯◯  LOW | Critical |

a: High I^2^ and discrepant results

**Additional file 2: Table S4** Meta-regression results

|  | **Quality of the covariates adjusted for** | **Geographical location** | **OHCA etiology** | **Type of cohort** | **Denominator of the population** | **Timeframe** |
| --- | --- | --- | --- | --- | --- | --- |
| *R^2^* (%) | 1.29 | 1.84 | 0 | 2.42 | 10.52 | 11.42 |
| p | 0.07 | 0.17 | 0.58 | 0.20 | 0.06 | 0.80 |
