# Supplementary material for: Sex and out-of-hospital cardiac arrest survival: a systematic review
Source: Ann Intensive Care. 2022 Dec 19;12:114. doi: 10.1186/s13613-022-01091-9 (PMC9763524; doi:10.1186/s13613-022-01091-9)
Supplement: Supplementary file 3 — Additional file 3: Database. [file 13613_2022_1091_MOESM3_ESM.docx]

**Additional file 3 -Database**

| **Study** | **Journal** | **Author** | **Location** | **Publication year** | **Period** | **Type of study and**  **Description** | **Source of data** | **Size of population covered** | **Denominator** | **Number of patients** | | **Patients survived to hospital discharge/30-day survival*** | | **OR (univariate)** | | **Adjusted survival to hospital discharge**  *** converted from in-hospital mortality** | | **Transformation of data with male as reference** | **Variables of adjustement** | **CPC 1-2 at discharge**  *** at 30-day** | | |
| --- | --- | --- | --- | --- | --- | --- | --- | --- | --- | --- | --- | --- | --- | --- | --- | --- | --- | --- | --- | --- | --- | --- |
|  |  |  |  |  |  | Retrospective, prospective, cohort. | N° of centres  Registry |  |  | Men | Women | Men | Women | Reference | OR (CI 95%) | Reference | OR |  |  | | Men | Women |
| **Ahn 2012** | American Journal of Emergency Medicine | Ki Ok Ahn | South Korea | 2012 | 2008 | retrospective observational analysis | National registry – CAVAS (South Korean registry of OHCA) | 49 million | All OHCA, presumed cardiac etiology | 8764 | 5158 | 274 | 94 | Female, survive to hospital discharge | 0.58 (0.45-0.73) | Female, discharge | 0.82 (0.63-1.05) |  | age, location of arrest, witness status, initial ECG rhythm, response time, and transport time | | Null | Null |
| **Akahane 2011** | American Journal of Medicine | Manabu Akahane | Japan | 2011 | 2005-2007 | retrospective observational analysis | National registry - Japanese registry of OHCA recorded by EMS. | 128 million | All OHCA | 171970 | 104620 | 9017* | 4478* | Female, 30-day survival | 0.81 (0.78-0.84) | Female, 1 month survival | 1.06 (1.02-1.1) |  | age, cause of arrest (cardiac/non cardiac), witness status, bystander CPR, defibrillation by EMS, AED by layperson, airway device, epinephrine | | 4285 | 1769 |
| **Al-Dury 2020** | American Journal of Emergency Medicine | Nooraldeen Al-Dury | Sweden | 2020 | 2011-2019 | retrospective observational analysis | National registry - Swedish Registry of Cardiopulmonary Resuscitation (Sweden’s reported OHCA by EMS) | 10 million | All OHCA | 14755 | 7024 | 1682* | 448* | Female,,  30-day survival | 0.53 (0.48-0.59) | Null | Null |  | Null | | Null | Null |
| **Allan 2016 POSTER** | Canadian Journal of Cardiology | KS Allan | Greater Toronto Area, Ontario, Canada | 2016 | 2009-2012 | retrospective observational analysis | Local Cohort - Registry of Greater Toronto Area | 5.9 million | All OHCA, presumed cardiac etiology | 340 | 110 | 76 | 37 | Female,, survive to hospital discharge | 1.76 (1.10-2.82) | Female, discharge | 2.92(1.44,5.91) |  | Age, rhythm, bystander witnessed, bystander CPR, Public location non-smoker hypertension, diabetes. | | Null | Null |
| **Arabi 2013 POSTER** | Journal of the American College of Cardiology | Abdulrahman Arabi | Doha, Qatar | 2013 | 1991-2010 | retrospective observational analysis | Single center - Registry of Hamad Medical Corporation Doha | 200k-650k | All OHCA | 718 | 269 | 304 | 93 | Female,, survive to hospital discharge | 0.72 (0.54-0.96) | Null | Null |  | Null | | Null | Null |
| **Arrich 2006** | Medicine | Jasmin Arrich | Vienna, Austria | 2006 | 1991-2004 | Local prospective database  retrospective analysis | Single center – Emergency department, University hospital in Vienna | 1.5 million | OHCA survivors resuscitated and admitted | 569 | 205 | 258 | 81 | Female,, survive to hospital discharge | 0.79 (0.57-1.09) | female, to discharge * | 0.91(0.60-1.38) |  | Age, BLS, No-flow and Low-flow time (minutes), Rhythm Medical History (myocardial Infarction, COPD, diabetes, hypertension, cerebral vascular disease, chronic heart failure), NYHA, Smoking, Use of therapeutic hypothermia | | Null | Null |
| **Auricchio 2020** | Resuscitation Plus | Angelo Auricchio | Ticino, Switzerland | 2020 | 2002-2018 | retrospective observational analysis | Local Cohort – registry of OHCA in Ticino canton, Switzerland | 350,000 | All OHCA, presumed cardiac etiology | 1788 | 693 | 341 | 80 | Female,, survive to hospital discharge | 0.55 (0.43-0.72) | Male, discharge | 1.13(0.8-1.5) | 0.88(0.66,-1.25) | age, presenting rhythm, year-groups of OHCA’s occurrence, OHCA location, EMS arrival time, witnessed status and CPR-initiated by laypeople | | 257 | 58 |
| **Blom 2019** | European Heart Journal | Blom, Marieke T | Amsterdam, the Netherlands | 2019 | 2006-2012 | retrospective observational analysis | Local Cohort -  ARREST (AmsteRdam REscustation STudies) registry of EMS treated patients | 2 million | All OHCA | 4117 | 1600 | 826 | 200 | Female, survive to hospital discharge | 0.57 (0.48-0.67) | Null | Null |  | Null | | Null | Null |
| **Bougouin 2017** | Resuscitation | Bougouin, Wulfran | Paris, France | 2017 | 2000-2013 | Local prospective database  retrospective analysis | Single center – PROCAT (Parisian Region Out of Hospital Cardiac ArresT) | 6.5 million | OHCA survivors resuscitated and admitted | 1297 | 520 | 442 | 150 | Female,, survive to hospital discharge | 0.78 (0.63-0.98) | male, discharge | 0.77(0.57 - 1.06) | 1.29(0.94,1.75) | Age, per year, Occurrence at home, Shockable Rhythm, Time from collapse to BLS > 4 min, Time from BLS to ROSC > 15 min, Epinephrine use, Early invasive strategy, Post-OHCA shock | | 418 | 138(ICU discharge) |
| **Bray 2013** | Resuscitation | Janet E. Bray | Victoria, Australia | 2012 | 2003-2010 | retrospective observational analysis | Local cohort -  VACAR (Victorian Ambulance Cardiac Arrest Registry) registry. | 5.5 million | All OHCA | 7345 | 3108 | 808 | 218 | Female, survive to hospital discharge | 0.61 (0.52-0.71) | Female to discharge | 1.11(0.92-1.33) |  | age, witnessed arrest, bystander CPR, year of arrest, rural location, public location, EMS response time and interaction term if significant + shockable rhythm | | Null | Null |
| **Castro 2019 POSTER** | Journal of the American College of Cardiology | Yulanka Castro | United States of America | 2019 | 2012-2016 | National registry  retrospective | National Registry -  National Inpatient Sample (USA database of inpatient care data) | 320 million | All OHCA | 533985 | 410175 | 218934 | 151765 | Female, survive to hospital discharge | 0.85 (0.84-0.85) | Female, to discharge * | 0.88(0.86-0.90) |  | acute kidney injury, ST-segment elevation myocardial infarction (STEMI), and cardiogenic shock | | Null | Null |
| **Cline 2002** | Heart Rhythm | Sharon L. Cline | Indiana, USA | 2002 | 1997-1999 | prospective | Local Cohort -  PARADE (Police as Responder Automated Defibrillation Evaluation) trial in 6 rural counties in Indiana, USA. | 465k | All OHCA | 250 | 138 | 18 | 3 | Female,  survive to hospital discharge | 0.29 (0.08-0.99) | Female, to discharge | 0.29 (0.08-0.99) |  | age | | Null | Null |
| **Dicker 2018** | Emergency Medicine Journal | Dicker, Bridget | New Zealand | 2018 | 2013-2015 | National registry  retrospective | Local cohort – SOCAR (St John New Zealand OHCA Registry) | 4 million | All OHCA | 2678 | 1184 | 432* | 148* | Female,  30-day survival | 0.74 (0.61-0.91) | Female, 30-day survival | 1.22 (0.96- 1.55) |  | age, location, aetiology, initial rhythm, witnessed status, PCI | | Null | Null |
| **Goto 2019** | Critical care | Yoshikazu Goto | Japan | 2019 | 2013-2016 | observational analysis  retrospective | National Registry -  FDMA (Fire and Disaster Management Agency) registry of EMS in Japan. | 127 million | All OHCA | 217173 | 169362 | 12373* | 5561* | Female,  30-day survival | 0.56 (0.54-0.58) | Female, 30-day survival | 1.07 (1.03-1.11) | 0.93(0.90, 0.97) | age, year, place, witness status, intial rhythm, presumed caused, bystander CPR, airway managment, epinephrine, call-to-response time\hospital arrival. | | 6936 (1m) | 2327 |
| **Herlitz 2004** | Resuscitation | Johan Herlitz | Sweden | 2004 | 1990-2000 | observational analysis  retrospective | Local cohort (data reported from 1990 when only few ambulance services were included in the registry) -  Swedish Cardiac Arrest Registry | 9 million | All OHCA | 17149 | 6648 | 514* | 199* | Female,  30-day survival | 1.00 (0.85-1.18) | Female, 30-day survival | 1.27 (1.03-1.56) |  | age, the witnessed status, bystander CPR, place of arrest, initial rhythm, and aetiology | | Null | Null |
| **Hubert 2020** | European Journal of Emergency Medicine | Hervé Hubert | France | 2020 | 2011-2017 | National registry  retrospective | National Registry -  RéAC (French National Cardiac Arrest Registry) | 66 million | All OHCA | 43655 | 22740 | 2575* | 978* | Female, 30-day survival | 0.72 (0.66-0.77) | Male, 30-day survival | 0.80(0.69-0.92) | 1.25(1.08-1.44) | age, cardiac arrest type, location, bystander presence, bystander type, cardiovascular history, respiratory history, diabetes history, end of life, and rhythm at MMT arrival | | 36888* | 18783* |
| **Jeong 2019** | The American Journal of Emergency Medicine | Jin Seop Jeong | South Korea | 2019 | 2013-2016 | observational study  retrospective | National Registry -  OHCA database of Korea | 48 million | All OHCA, presumed cardiac etiology | 13716 | 6959 | 3795 | 1163 | Female, survive to hospital discharge | 0.52 (0.49-0.56) | female, discharge | 0.87 (0.71-1.08) |  | patient-community/EMS factors (Model 1 + comorbidities, metropolitan, place of arrest, witness, bystander CPR, EMS defibrillation, EMS response time, primary cardiac rhythm at the scene, and time from EMS call to ROSC +hypothermia and level of ED + PCI | | 2420 | 587 |
| **Johnson 2013** | Resuscitation | M. Austin Johnson | American | 2012 | 2005-2009 | observational analysis  retrospective | Local Cohort -  CARES (Cardiac Arrest Registry to Enhance Survival) registry of 29 cities across the USA | 22 million | All OHCA | 11745 | 7653 | 1159 | 588 | Female, survive to hospital discharge | 0.76 (0.69-0.84) | Female to discharge | 1.23 (1.09–1.38) |  | Age, Race/ethnicity, Public arrest Witnessed by bystander Witnessed by EMS Bystander CPR Public AED used Shockable rhythm | | Null | Null |
| **Kim 2001** | Circulation | Catherine Kim | Washington, USA | 2001 | 1990-1998 | cohort  retrospective | Local Cohort –  Cardiac arrests in King County and Seattle, Washington, USA. | 1 million | All OHCA | 7069 | 3810 | 1056 | 403 | Female, survive to hospital discharge | 0.67 (0.60-0.76) | female, to discharge | 1.09 (0.93 to 1.27) |  | age, vf, witnessed arrest, bystander CPR, categorized location of arrest, response time by first responder, and response time by paramedic | | Null | Null |
| **Mahapatra 2003** | Resuscitation | S. Mahapatra | Olmsted County, MN, USA | 2003 | 1990-2000 | observational analysis  retrospective | Local Cohort –  Olmsted County, Minnesota, USA registry of VF OHCA | 140,000 | VF OHCA | 163 | 37 | 66 | 13 | Female, survive to hospital discharge | 0.80 (0.38-1.68) | Null | Null |  | Null | | Null | Null |
| **Nagraj 2022**  **POSTER** | Journal of the American College of Cardiology | Sanjana Nagraj | New York City, NY, United States | 2022 | 2019-2021 | observational analysis  retrospective | Single center –  New York City  Health, Hospitals/Jacobi OHCA admission |  | OHCA survivors resuscitated and admitted | 80 | 74 | 15 | 11 | Female, survive to hospital discharge | 0.76 (0.32-1.77) | Reference not mentioned, unexploitable results | Null |  | Age, race, witnessed arrest, bystander CPR, Initial shockable rhythm, EMS response time, location of arrest, resuscitation duration | | Null | Null |
| **Ng 2016** | Resuscitation | Yih Yng Ng | 7 countries in Asia | 2016 | 2009-2012 | observational analysis  retrospective | International Registry -  PAROS (Pan-Asian Resuscitation Outcomes Study) registry of 12 sites in 7 Asian countries (Singapore, Japan, South Korea, Malaysia, Thailand, Taiwan and United Arab Emirates. |  | All OHCA | 24267 | 15892 | 1936 | 631 | Female, survive to hospital discharge | 0.48 (0.43-0.52) | Female, to discharge | 0.94 (0.77-1.15) |  | age, gender, location type, medical history, arrest witnessed status, bystander CPR, initial arrest rhythm, prehospital defibrillation, prehospital airway, prehospital drug administration, response time | | 1172 | 318 |
| **Pell 2000** | European Heart Journal | J. P. Pell | Scotland | 2000 | 1988-1997 | Prospective cohort study | National Registry -  Scotland national registry | 5 million | All OHCA | 15437 | 6724 | 1113 | 422 | Female, survive to hospital discharge | 0.86 (0.77-0.97) | female, to discharge | 0.96 (0.8-1.14) |  | age, gender, defibrillation, arrest location, witness, time to CPR | | Null | Null |
| **Perman 2019** | Clinical Therapeutics | Perman, Sarah M | California, USA | 2019 | 2010-2011 | cross-sectional analysis  retrospective | National Registry –  SID (California State Inpatient Dataset) registry of OHCA in CA | 37 million | All OHCA | 3675 | 2887 | 1513 (patients minus died during hospitalization) | 1048 (patients minus died during hospitalization) | Female, survive to hospital discharge | 0.81 (0.74-0.90) | Female, to discharge (converted from mortality) | 0.86 (0.77-0.96) |  | age, race, and chronic medical conditions | | Null | Null |
| **Rob 2022** | American Journal of Emergency Medicine | Daniel Rob | Prague, Czech Republic | 2022 | 2012-2020 | retrospective observational analysis | Single center –  General University Hospital in Prague, Czech Republic admissions of OHCA. | 1.3 million | All OHCA | 693 | 239 | 369* | 105* | Female, 30-day survival | 0.69 (0.51-0.92) | Null | Null |  | Null | | Null | Null |
| **Safdar 2014** | Academic Emergency Medicine | Basmah Safdar | Ontario, Canada | 2014 | 1994-2002 | retrospective observational analysis | Local Cohort –  OPALS (Ontario Prehospital Advanced Life Support) registry | 12 milion | All OHCA | 7748 | 3731 | 248 (converted from percentages) | 63 (converted from percentages) | Female, survive to hospital discharge | 0.52 (0.39-0.69) | Female survival to hospital discharge | 0.88 (0.81–0.96) |  | age, witnessed arrest, initial rhythm upon EMS arrival, provision of bystander CPR, type of ALS (BLS, BLS optimisation, ALS) | | Null | Null |
| **Shin 2010**  **POSTER** | Circulation | Sang Do Shin | South Korea | 2010 | 2006-2007 | retrospective observational analysis | National registry –  CAVAS (South Korean registry of OHCA) | 49 million | All OHCA, presumed cardiac etiology | 12111 | 6934 | 392 | 131 | Female, survive to hospital discharge | 0.58 (0.47-0.70) | Null | Null |  | Null | | Null | Null |
| **Wissenberg 2014** | Resuscitation | Mads Wissenberg | Denmark | 2014 | 2001-2010 | retrospective observational analysis | National registry –  Denmark’s registry | 5.6 million | All OHCA,  presumed cardiac etiology | 13054 | 6318 | 1033* | 277* | Female,, 30-day survival | 0.53 (0.47-0.61) | Null | Null |  | Null | | Null | Null |
