# Supplementary material for: Sex and out-of-hospital cardiac arrest survival: a systematic review
Source: Ann Intensive Care. 2022 Dec 19;12:114. doi: 10.1186/s13613-022-01091-9 (PMC9763524; doi:10.1186/s13613-022-01091-9)
Supplement: Supplementary file 4 — Additional file 4: Figure S3. Quality of the variables used for adjustment: subgroup analysis of adjusted data on men and women survival to discharge or to 30 days after OHCA. Figure S3. BIS. Quality of the variables used for adjustment: subgroup analysis of adjusted data on men and women survival to discharge or to 30 days after OHCA (with the pooled τ2). Figure S4. Geographical location: subgroup analysis of adjusted data on men and women survival to discharge or to 30 days after OHCA. Figure S4 BIS. Geographical location: subgroup analysis of adjusted data on men and women survival to discharge or to 30 days after OHCA (with the pooled τ2). Figure S5. OHCA etiology: subgroup analysis of adjusted data on men and women survival to discharge or to 30 days after OHCA. Figure S6. Type of cohort and number of centres: subgroup analysis of adjusted data on men and women survival to discharge or to 30 days after OHCA. Figure S6. BIS Type of cohort and number of centres: subgroup analysis of adjusted data on men and women survival to discharge or to 30 days after OHCA (with the pooled τ2). Figure S7. Population denominator (whether non-survivors to hospital admission were included or not): subgroup analysis of adjusted data on men and women survival to discharge or to 30 days after OHCA. Figure S8. Scatter plot showing the results of the regression of time vs. the log(OR) for adjusted survival to discharge or to 30 days. A log(OR) below 0 favors men and a log(OR) above 0 favors women. Figure S9. A Baujat plot showing the contribution of each study to the overall heterogeneity. B Same plot zoomed in Studies are numbered in alphabetical order. Figure S10. Sensitivity analysis omitting studies rated identified as outliers in the Baujat plot (see Fig. 9)—adjusted survival data. Figure S11. Sensitivity analysis omitting studies including traumatic etiology for OHCA—adjusted survival data. Figure S12. Sensitivity analysis omitting studies assessed as having a high risk of bias. [file 13613_2022_1091_MOESM4_ESM.docx]

**Additional file 4 – Meta-analysis**

The first subgroup analysis was based on the covariates adjusted for. Based on prior associations observed between the covariates and patient outcomes in the literature, each covariate was classified as “strong” (initial rhythm and bystander CPR), “medium” (age and etiology) or “weak” (other variables). Initially, significant differences were found between the subgroups in the effect-size (p<0.001, Fig.3). However, when the small number of studies in the “weak” and “medium” subgroups was taken into account, no credible subgroup effects were observed (p=0.07, Fig.3 BIS).

The second subgroup analysis was based on the geographical location of the cohort. No credible subgroup effects were identified either with (p=0.18, Fig. 4 BIS) or without (p=0.10, Fig. 4) taking into consideration the small number of studies in “Oceania” and “Asia” groups.

The third subgroup analysis was based on the etiology of the OHCA. Nine studies included only OHCAs from cardiac origin, while twelve studies included both cardiac and non-cardiac OHCAs. The results showed no statistical differences in the effect-size between groups (p=0.71, Fig. 5).

A subgroup analysis was also performed according to the type of cohort (national databases, local cohorts based on EMS data, international cohorts based on EMS data and monocentric cohorts). The results showed no statistical differences in the effect-size between groups either with (p=0.21, Fig 6 BIS) or without (p=0.27, Fig. 6) taking into consideration the small number of studies in “monocentric” and “international cohort from EMS data” groups.

Subgroup analysis based on the denominator of the population (*i.e.* whether non-survivors to hospital admission were accounted for the cohort or not) was conducted. The results showed significant differences in the effect-size between groups (p<0.01, Fig.7).

Finally, meta-regression analyses were performed for each subgroup yielding non-significant results (e-Table 4). While a significant difference was found in the subgroup analysis regarding the population denominator, the meta-regression failed to show any significant participation of this variable to the effect size (*R^2^*=10.52%, p=0.06, e-Table 4). We also assessed the effect of time on heterogeneity by performing a meta-regression of the OR of survival *versus* the last year of patient inclusion in the cohort. The meta-regression analysis showed little non-significant involvement of time in heterogeneity with an *R^2^*=11.42% and p=0.80 (e-Table 4). The results are presented in a scatter plot as Figure 8.

*Sensitivity analyses*

As subgroup analyses failed to identify causes for the bulk of the heterogeneity, a Baujat plot was performed to identify outlier studies (Fig. 9). External inspection of the plot identified six outlier studies (Castro, Akahane, Johnsonn, Hubert, Allan, Herlitz). Omission of these studies from the analysis led to a decrease in overall heterogeneity with an OR of adjusted survival for women 0.97 [0.91-1.04] and an *I^2^*=55% (Fig. 10).

A sensitivity analysis was also performed to restrain the analysis to studies including only non-traumatic OHCA. Omission of the studies including traumatic OHCA did not lead to a significant decrease in the heterogeneity, with an OR adjusted survival for women 1.01 [0.90-1.15], *I^2^*=75% (Fig 11).

Further sensitivity analyses were performed, with the omission of studies displaying studies in a non-Utstein style, or studies not using dedicated cardiac arrest databases (Castro and Perman used national inpatient databases in the US), yielding inconclusive results (1.01 [0.93-1.09], *I^2^*=79% and 1.04 [0.97-1.12], *I^2^*=79% respectively).

Due to the risk of collinearity between adjustment variables and sex, we decided to perform a sensitivity analysis omitting studies with adjustment variables related to care management, which lead to no significant change in the effect size or the statistical heterogeneity (OR 1.07 [0.93-1.23], *I^2^*=88%).

Finally, a sensitivity analysis based on the risk of bias assessment was performed. Studies assessed as having a high risk of bias were omitted, leading to an non-significant decrease in heterogeneity (OR 1.01 [0.98-1.12], *I^2^*=80%, Fig.12).

*Secondary outcomes*

*Unadjusted survival* - Unadjusted data on survival was reported in twenty-eight studies: seven of them reported 30-day survival and twenty-one studies reported survival to discharge. Females had a lower likelihood of survival than males, with an OR 0.68 [0.62-0.74], PI [0.43-1.05], *I^2^*=97% (Fig.13). Of note, the value of no difference was included in the PI, there was therefore weak evidence to support a significative difference between sexes in future studies with similar characteristics. Inspection of the funnel plot suggested a publication bias by showing an asymmetry, this was however not confirmed by the Rücker’s test (p= 0.058) (Fig.14).

*Favorable neurological outcome* - Unadjusted data on favorable neurological outcome was reported in twelve studies. Men had a significantly higher likelihood of favorable neurological outcome that did women with an OR 0.56 [0.49-0.66], PI [0.31-1.03], *I^2^*=95% (Fig. 15). Of note, the value of no difference was included in the PI, there was therefore weak evidence to support a significative difference between sexes in future studies with similar characteristics.

This trend disappeared when the data was adjusted, with no difference between men in women in adjusted favorable neurological outcome (number of studies: seven, OR 0.96 [0.83-1.10], *I^2^*=84%) (Fig. 16).

**Figure S3.** Quality of the variables used for adjustment: subgroup analysis of adjusted data on men and women survival to discharge or to 30-day after OHCA.

**Figure S3. BIS.** Quality of the variables used for adjustment: subgroup analysis of adjusted data on men and women survival to discharge or to 30-day after OHCA (*with the pooled τ^2^*)

**Figure S4.** Geographical location: subgroup analysis of adjusted data on men and women survival to discharge or to 30-day after OHCA

**Figure S4 BIS.** Geographical location: subgroup analysis of adjusted data on men and women survival to discharge or to 30-day after OHCA (*with the pooled τ^2^*)

**Figure S5.** OHCA etiology: subgroup analysis of adjusted data on men and women survival to discharge or to 30-day after OHCA

**Figure S6.** Type of cohort and number of centres: subgroup analysis of adjusted data on men and women survival to discharge or to 30-day after OHCA

**Figure S6. BIS** Type of cohort and number of centres: subgroup analysis of adjusted data on men and women survival to discharge or to 30-day after OHCA (*with the pooled τ^2^*)

**Figure S7.** Population denominator (whether non-survivors to hospital admission were included or not): subgroup analysis of adjusted data on men and women survival to discharge or to 30-day after OHCA

**Figure S8.** Scatter plot showing the results of the regression of time versus the log(OR) for adjusted survival to discharge or to 30-day. A log(OR) below 0 favors men and a log(OR) above 0 favors women.

B

A

**Figure S9. A.** Baujat plot showing the contribution of each study to the overall heterogeneity**B.** Same plot zoomed in Studies are numbered in alphabetical order.

N°8 Castro, n°2 Akahane, n°15 Johnsonn, n°13 Hubert, n°3 Allan, and n°12 Herlitz were considered as outliers

**Figure S10**. Sensitivity analysis omitting studies rated identified as outliers in the Baujat plot (see Figure 9) - Adjusted survival data

**Figure S11**. Sensitivity analysis omitting studies including traumatic etiology for OHCA - Adjusted survival data

**Figure S12**. Sensitivity analysis omitting studies assessed as having a high risk of bias

**Figure S13.** Forest plot of unadjusted data on men and women survival to discharge or to 30-day after OHCA.

**Figure S14.** Funnel plot of the included studies

**Figure S15.** Forest plot of unadjusted data on men and women neurologically intact survival

**Figure S16.** Forest plot of adjusted data on men and women favourable neurological outcome in survival
